# Hemodynamic variations and anxiety during the surgical extraction of impacted lower third molars

**DOI:** 10.4317/jced.55294

**Published:** 2019-01-01

**Authors:** Pablo Tarazona-Álvarez, Hilario Pellicer-Chover, Beatriz Tarazona-Álvarez, David Peñarrocha-Oltra, María Peñarrocha-Diago

**Affiliations:** 1 DDS. Master in Oral Surgery and Implantology. University of Valencia Medical and Dental School; 2 DDS, PhD. Assistant Professor of Orthodontics. University of Valencia Medical and Dental School; 3 DDS, PhD. Assistant Professor of Oral Surgery and Implantology. University of Valencia Medical and Dental School; 4 Full Professor of Oral Surgery. Professor of the Master in Oral Surgery and Implantology. University of Valencia Medical and Dental School. Valencia, Spain

## Abstract

**Background:**

The surgical removal of an impacted third molar can cause patient anxiety. Such anxiety and the use of vasoconstrictor drugs and local anesthetics in turn can induce hemodynamic variations during the operation. A study is made of the variations in hemodynamic parameters (systolic and diastolic blood pressure and heart rate) and their correlation to patient gender and anxiety during surgical removal of an impacted lower third molar.

**Material and Methods:**

A prospective study was carried out in the Oral Surgery Unit of a university clinic, with the inclusion of 125 patients (mean age 24.9 years). Anesthesia was administered in the form of 4% articaine and adrenalin 1:200,000 for surgical removal of the impacted lower third molars.

**Results:**

Women experienced greater anxiety than men. Systolic blood pressure showed few changes – the maximum and minimum values being recorded at the time of incision and upon suturing, respectively. Diastolic blood pressure in turn showed maximum and minimum values before the start of surgery and during extraction, respectively, while heart rate proved maximum during incision and minimum upon suturing. The differences in systolic and diastolic blood pressure, and heart rate, between men and women, and between patients with and without anxiety, failed to reach statistical significance.

**Conclusions:**

The fact that these were young patients could contribute to explain the absence of significant hemodynamic changes in our study.

** Key words:**Anxiety, third molars, extraction, surgery.

## Introduction

The vasoconstrictor drugs and local anesthetics used in oral surgery can induce hemodynamic variations during the removal of an impacted lower third molar, in the same way as other factors such as patient stress or anxiety (1). However, some authors consider that blood pressure elevation during surgery is not attributable to the vasoconstrictor used with the anesthetic agent but to anxiety or other factors in inherent to the patient (2).

The etiology underlying patient dental anxiety comprises a range of factors, including congenital aspects (3), previous trauma (4,5) or the past experiences of relatives or friends (4,5). Such patient anxiety is observed in situations implying anesthetic injections, the use of rotary instruments and/or tooth extractions (7-10). The surgical removal of an impacted third molar is a clear example of this kind of situation.

The present study was carried out to analyze the hemodynamic variations (systolic [SBP] and diastolic blood pressure [DBP], and heart rate [HR]) and their correlation to patient gender and anxiety during the surgical removal of an impacted lower third molar using anesthesia in the form of 4% articaine and adrenalin 1:200,000.

## Material and Methods

-Study sample

A prospective clinical study was carried out in the Oral Surgery Unit of a university hospital. All patients gave informed consent, and the study was approved by the Ethics Committee of the University of Valencia (Valencia, Spain)(Ref. H1335344183371). From among the total of 206 patients requiring the removal of impacted third lower molars between September 2010 and January 2011, we selected those who were adults; had not undergone previous impacted lower third molar surgery; presented an ASA score I or II (11); and required flap elevation, ostectomy and tooth sectioning.

We excluded patients with allergy to local anesthetics or to any other drug; pregnant or nursing women; individuals with HR > 110 bpm or < 60 bpm; patients with SBP > 150 mmHg or < 100 mmHg and DBP > 100 mmHg or < 60 mmHg; individuals with pain, inflammation or signs of infection in the zone of the third molar; patients receiving any kind of medication in the 15 days before surgery; and surgeries lasting less than 15 minutes or more than 45 minutes (12). Of the selected patients, 24 with incomplete information were excluded from the study. The final sample consisted of 125 patients (53 men and 72 women) with a mean age of 24.9 years (range 18 – 52).

-Surgical procedure

The patients underwent lingual and inferior alveolar nerve block administering 1.8 ml of 4% articaine (Ultracain®, Inibsa, Barcelona, Spain) with a vasoconstrictor (adrenalin 1:200,000) using a 27G needle measuring 35 mm in length (Sofic® XL Monoprotect, France). Supplemental anesthesia of the buccal nerve was added 5 minutes after the first injection, administering 1.8 ml of a second carpule containing the same solution with a 30G needle measuring 25 mm in length (Sofic® XL Monoprotect, France).

The operation was carried out by two dentists with the same surgical experience, in the same operating room and under the same working conditions. A distal incision with a vertical releasing incision mesial to the second molar was performed, with mucoperiosteal detachment followed by ostectomy and tooth sectioning. After removal of the third molar, the mucoperiosteal flap was repositioned with 3/0 braided silk sutures (Lorca Marin®, Murcia, Spain). The sutures were removed one week after surgery.

-Data collection

Immediately before the operation, the patients completed Corah’s Dental Anxiety Scale (DAS) ( Table 1). The DAS (13) comprises four questions with 5 possible answers, and is scored from 1 (relaxed) to 5 (almost physically ill), yielding a range of scores between 4-20 points. The absence of anxiety was defined by a score of < 12 points, while anxiety was defined as a score of ≥ 12 points.

**Table 1:**
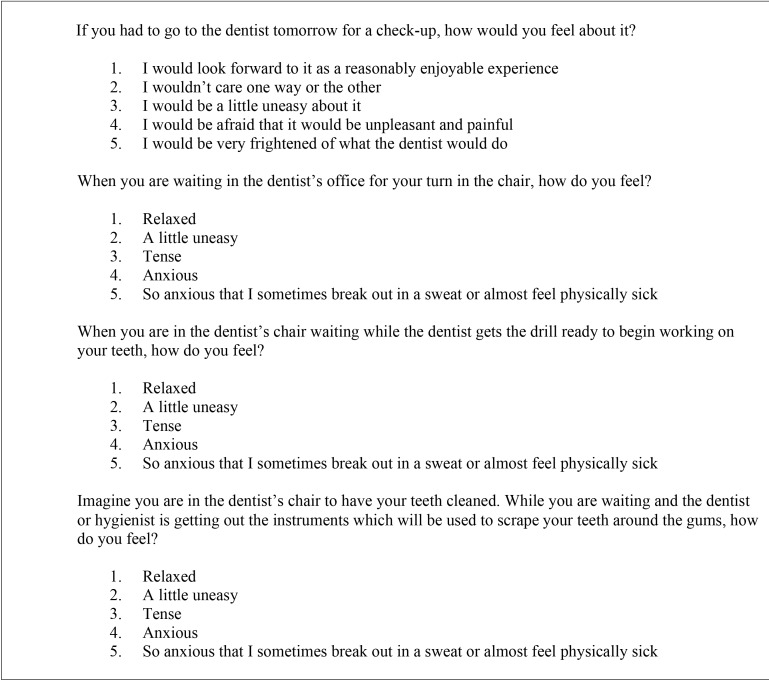
Corah’s Dental Anxiety Scale (DAS).

We likewise recorded the duration of surgery (defined as the time from incision to the end of suturing) and surgical difficulty using the scale of Alemany-Martínez *et al.* (14)(scored as minimally, moderately or very difficult). Intraoperative pain was recorded at the end of treatment based on a visual analog scale (VAS) from 0-100 mm.

Systolic and diastolic blood pressure and heart rate were recorded using a tensiometer (OMRON M6, HEM-7001-E, Paris, France), at the following timepoints: before surgery (A1) and following anesthesia (A2), incision (A3), ostectomy (A4), extraction (A5) and suturing (A6).

-Statistical analysis

A univariate descriptive analysis was made, reporting the data as percentages and frequencies. Analysis of variance (ANOVA) for repeated measures was used for the comparative study. The SPSS version 15 statistical package (SPSS Inc., Chicago, IL, USA) was used throughout. Statistical significance was considered for *p* < 0.05 in all cases.

## Results

Before the operation, the mean DAS score among the patients without anxiety (n=77) was 8.9 points, while that of the anxious patients (n=48) was 13.5 points. With regard to gender, women showed greater anxiety (DAS= 10.5) than men (DAS= 7.4) (*p*=0.00).

The mean duration of surgery was 30.2 ± 10.7 minutes. Surgical difficulty as determined by the scale of Alemany-Martínez *et al.* (14) proved minimum in 95 teeth and moderate in 30. The intraoperative pain score was 3.2 in men and 2.6 in women – the difference being nonsignificant (*p*=0.104).

 Table 2 reports the mean SBP, DBP and HR values at the different surgical timepoints. Systolic blood pressure showed few changes – the highest value corresponding to the time of incision and the lowest to the time of suturing. In turn, DBP was highest before the operation and lowest during extraction, while HR was highest during incision and lowest at the time of suturing.

**Table 2:**
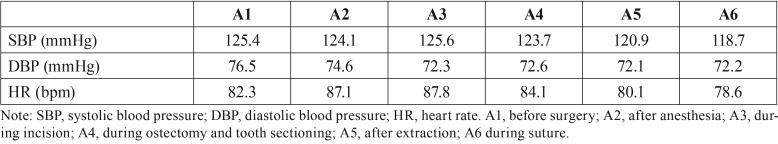
Mean systolic (SBP) and diastolic blood pressure (DBP) values and heart rate (HR) at the different data collection timepoints.

-Systolic blood pressure

**Figure 1:**
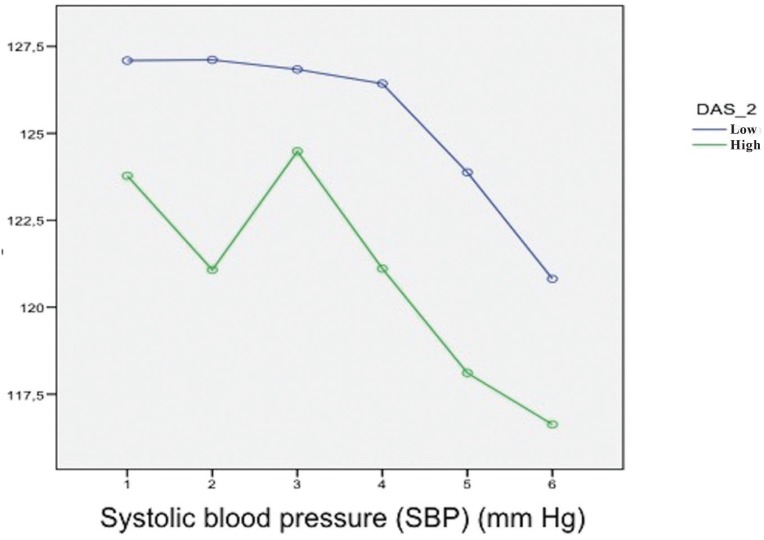
Correlations between the hemodynamic changes during the surgical procedure and anxiety scored with the Dental Anxiety Scale (DAS). Levels of anxiety: high and low. Systolic blood pressure (SBP) and Dental Anxiety Scale (DAS).

The mean SBP during surgery was 124.9 ± 1.4 mmHg. The distribution according to gender was 128.4 mmHg in men and 121.4 mmHg in women – the difference being nonsignificant (*p*=0.336). Both men and women experienced a gradual decrease in SBP up to the time of suturing ( Table 3). Patients experiencing anxiety presented a SBP of 120.9 ± 2.2 mmHg, while the patients without anxiety presented a lower value of 125.4 ± 1.2 mmHg – the difference being nonsignificant (*p*=0.502) (Fig. 1).

**Table 3:**
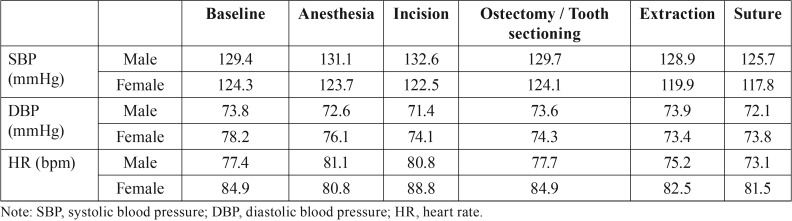
Hemodynamic changes during the surgical procedure according to patient gender. Systolic and diastolic blood pressure expressed in mmHg and heart rate as bpm.

-Diastolic blood pressure

**Figure 2:**
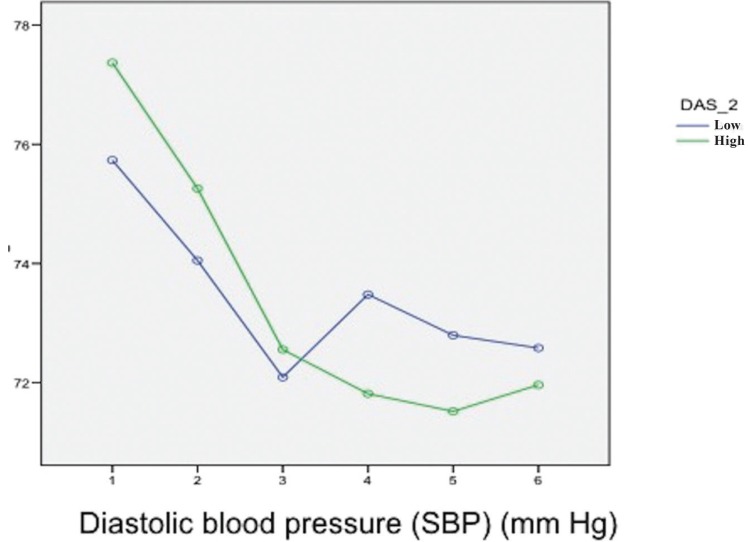
Correlations between the hemodynamic changes during the surgical procedure and anxiety scored with the Dental Anxiety Scale (DAS). Levels of anxiety: high and low. Diastolic blood pressure (DBP) and Dental Anxiety Scale (DAS).

The mean DBP during surgery was 75.2 ± 1.5 mmHg. The distribution according to gender was 72.3 mmHg in men and 74.2 mmHg in women – the difference being marginally significant (*p*=0.045). Both men and women experienced a decrease in DBP from baseline up to the time of incision. From this timepoint the values were similar until the end of the operation ( Table 3).The mean DBP was 73.4 mmHg in both patients with and without anxiety (*p*=0.525) (Fig. 2).

-Heart rate

**Figure 3:**
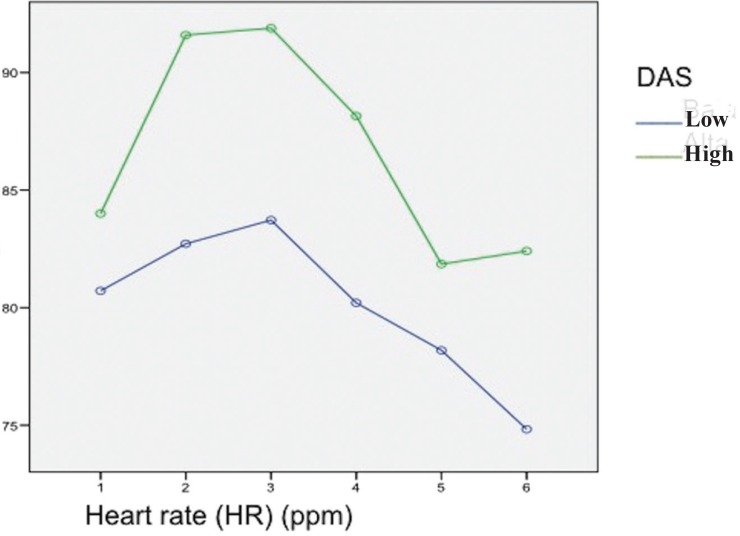
Correlations between the hemodynamic changes during the surgical procedure and anxiety scored with the Dental Anxiety Scale (DAS). Levels of anxiety: high and low. Heart rate (HR) and Dental Anxiety Scale (DAS).

The mean HR during surgery was 80.8 ± 1.5 bpm. The distribution according to gender was 84.7 ± 1.6 bpm in men and 77.1 ± 1.4 bpm in women – the difference being nonsignificant (*p*=0.884). The heart rate was seen to increase up to the time of incision, which is when the maximum values were recorded, followed by a decrease until the end of surgery ( Table 3).The patients with anxiety showed a mean HR of 86.6 ± 2.4 bpm, versus 80 ± 1.2 bpm in those without anxiety – the difference being nonsignificant (*p*=0.08) (Fig. 3).

## Discussion

In the present study, women yielded significantly higher dental anxiety scores than men (*p*=0.00) ( Table 3) – this being consistent with the observations of most studies found in the literature (15,16). With regard to intraoperative pain, the scores were seen to be lower in women than in men. Fagade *et al.* (17) likewise found men to have a lower pain threshold than women, and the latter were more tolerant of pain. However, other authors (18) have found men to experience less pain than women during the surgical procedure. In any case, gender differences in the experience of pain have been reported by a number of investigators (19,20).

In our study involving normotensive patients, both SBP and HR increased in men until the time of incision (A3), while DBP decreased up until incision in both genders. Our findings are not consistent with those of other studies (2,21) in which dental avulsion was seen to be the phase with the highest stress levels. According to Nichols (22), blood pressure is always highest at the start of the surgical procedure, as a result of endogenous adrenalin release caused by patient anxiety or fear of visiting the dentist. Men showed higher SBP values than women, while the latter had higher DBP and HR values and suffered greater anxiety. The patients with the highest anxiety scores had greater blood pressure values, though with lower HR – a circumstance that may be explained in terms of vasovagal syncope, as reported by Liau *et al.* (23).

Changes in blood pressure and HR can be influenced by pain and patient-related factors such as age, gender, hypertension, previous experience with dental treatments, and psychological response (24). The mean age of our patients was 24.9 years (range 18-52). The fact that these patients were young may help explain the absence of significant hemodynamic changes in our study. In comparison, Matsumura *et al.* (25) found middle-aged patients to experience greater blood pressure increments during oral surgery than younger individuals. Further studies involving older patients are needed to discard the existence of hemodynamic variations conditioned to gender and anxiety during the extraction of lower third molars.

## References

[1] Fukayama H, Yagiela JA (2006). Monitoring of vital signs during dental care. Int Dent J.

[2] Silvestre FJ, Verdú MJ, Sanchís JM, Grau D, Peñarrocha M (2001). Effects of vasoconstrictors in dentistry upon systolic and diastolic arterial pressure. Med Oral.

[3] Jackson E (1974). Managing dental fears: a tentative code of practice. J Oral Med.

[4] Berggren U, Meynert G (1984). Dental fear and avoidance: causes, symptoms, andconsequences. J Am Dent Assoc.

[5] Kleinknecht RA, Keplac RK, Alexander LD (1973). Origins and characteristics of fear of dentistry. J Am Dent Assoc.

[6] Scott DS, Hirschmann R, Schroder K (1984). Historical antecedents of dental anxiety. JADA.

[7] Molin C, Seeman K (1970). Disproportional dental anxiety: Clinical and nosological considerations. Acta Odontol Scand.

[8] Gale EN (1972). Fears of the dental situation. J Dent Res.

[9] Beggren U, Meynert G (1984). Dental fear and avoidance: Causes, symptoms and consequences. J Am Dent Assoc.

[10] Earl P (1994). Patient's anxieties with third molar surgery. Br J Oral Maxillofac Surg.

[11] McCarthy FM, Malamed SF (1979). Physical evaluation system to determine medical risk and indicated dental therapy modifications. J Am Dent Assoc.

[12] Meyer FU (1987). Hemodynamic changes under emotional stress following a minor surgical procedure under local ansthesia. Int J Oral Maxillofac Surg.

[13] Corah N, Gale E, Illig S (1978). Assessment of a dental anxiety scale. J Am Dent Assoc.

[14] Alemany-Martinez A, Valmaseda-Castellon E, Berini-Aytés L, Gay-Escoda C (2008). Hemodynamic changes during the surgical removal of lower third molars. J Oral Maxillofac Surg.

[15] Lago L, Diniz M, Senra C, Seoane G, Gándara J, García A (2006). Dental anxiety before removal of a third molar and association with general trait anxiety. J Oral Maxillofac Surg.

[16] Garip H, Abali O, Göker K, Göktürk Ü, Garip Y (2004). Anxiety and extraction of third molars in Turkish patients. Brit J Oral Maxillofac Surg.

[17] Fagade OO, Oginni FO (2005). Intraoperative pain perception in tooth extraction: Possible causes. Int Dent J.

[18] Colorado-Bonnin M, Valmaseda-Castellón E, Berini-Aytés L, Gay-Escoda C (2006). Quality of life following lower third molar removal. Int J Oral Maxillofac Surg.

[19] Robison ME, Gagnon CM, Dannecker EA,  Brown JL,  Jump RL,  Price DD (2003). Sex differences in common pain events: Expectations and anchors. J Pain.

[20] Robison ME, Gagnon CM,  Riley JL 3rd,  Price DD (2003). Altering gender role expectations: Effects on pain tolerance, pain threshold, and pain ratings. J Pain.

[21] Paramaesvaran M, Kingon AM (J 1994). Alterations in blood pressure and pulse rate in exodontia patients. Aust Dent.

[22] Nichols C (1997). Dentistry and hypertension. J Am Dent Assoc.

[23] Liau FL, Kok SH, Lee JJ, Kuo RC, Hwang CR, Yang PJ (2008). Cardiovascular influence of dental anxiety during local anesthesia for tooth extraction. Oral Surg Oral Med Oral Pathol Oral Radiol Endod.

[24] Brand HS, Abraham-Inpijn L (1996). Cardiovascular responses induced by dental treatment. Eur J Oral Sci.

[25] Matsumura K, Miura K,  Takata Y,  Kurokawa H,  Kajiyama M,  Abe I (1998). Changes in blood pressure and heart rate variability during dental surgery. Am J Hypertens.

